# Her Heart's Mistake

**Published:** 1889-04-06

**Authors:** E. D. Cumming

**Affiliations:** Author of "An Ordeal by Silence"


### HER HEART'S MISTAKE.

By E. D. Cumming, Author of "An Ordeal by Silence."

### Chapter I.—The New Arrival.

One more long, listless Indian day is drawing near its close, and the little world of Meerut is beginning to awaken from its torpor.

It is early in the month of June, and the hot season is almost at its height. The thermometer has never registered less than 105deg. in the coolest, most carefully darkened bungalow in the station since noon. It will rise yet higher ere the longed-for advent of the rains; but now the century has been passed, the perspiring observer regards the instrument's conduct with indifference. A few degrees more or less matter little, for there is no perceptible change in the steady, glaring heat. The airily attired sahib rouses himself to shout an irritable reminder to his prostrate coolie, and having startled that most languid of native servants into spasmodic activity, sinks wearily back in his long armchair again. Therein he dozes over his book all day, or dreams envious dreams of happy mortals up in the hills—those faraway havens of rest where the inhabitants enjoy all the beauties and suffer none of the vagaries of an English summer.

No breath of wind has arisen to stir the dusty leaves, and the still air outside strikes hot and dry upon the face, like the atmosphere of a furnace. The intense death-like silence which has reigned since noon is now disturbed by the chirrup of the little grey squirrel and the chattering of birds; for the sun, like a red, angry eyeball, is nearing the horizon, and the shadows cast by the mango trees are growing blurred and indistinct with the waning day.

On the Mall, the broad, shady road which runs through Meerut cantonments, and is to the residents what the Mile is to London society, the lank cheestus are busy with their water-skins laying the dust, for the white ladies and gentlemen will soon be abroad taking their evening drive or ride. Punjaubee policemen appear stealthily from mysterious nooks, adjusting belts and turbans with a guilty air, while they try to look as though they had not closed their eyes all day; their smothered yawns do suggest that they have indulged in a siesta, but now they are all alertness personified, and no stray goat dare show itself on the Mall without risk of being seized and impounded.

The shadows grow longer and fainter, and signs of animation increase. Strings of ponies, led by their syces, emerge from byeways and take the road to the polo ground near the railway station; half-a-dozen dusky youths set to work and make believe to sweep the tennis courts opposite the club. For whatever the temperature, young European India will not forego the active amusements to which this last precious hour of daylight is consecrated.

In every verandah white-robed servants are flitting to and fro, drawing up heavy green blinds and thick screens of kuskus grass. Nowhere are they busier than in the large bungalow at the corner where Wheeler-road joins the Mall. European houses in Meerut—indeed, throughout all Northern India—are externally much alike, and a short description of this residence, wherein the heroine of our story dwells, may serve to give the reader some idea of a fellow-countryman's home in this country.

It is a long, low whitewashed building, one storey high, thickly roofed with dark brown thatch, which forms an unbroken sheer from comb to eaves. Above the roof rise two stunted white chimney-stacks guiltless of "pots," which look strangely out of place for eight months in the year. A wide verandah runs all round the house, its cement floor raised a couple of feet or so from the level of the ground. The spaces between the whitewashed brick pillars which support the extreme lower edge of the roof are filled in with trellis-work arches, overgrown with luxuriant creepers, amongst whose foliage the bulbul and the ubiquitous sparrow build their nests. The bungalow stands back from the road, in its own compound or garden, and is shaded by a few tall mango trees now laden with unripe fruit. In the stone-bordered flower beds a few parched shrubs raise their heads from the sun-cracked soil, and give scanty promise of blossom when the south-west monsoon, with its dark rain clouds, shall come in July to renew their lease of life. The compound is enclosed by an open-work wall of bricks, among whose apertures the squirrels love to play hide-and-seek. And, finally, on the gatepost—there is no gate—hangs a board which bears the legend, "Surgeon-Major Harrison, A.M.D."

Dr. Harrison, in charge of the Army Medical Staff at Meerut, occupies this bungalow, together with his widowed sister, Mrs. Brent, and his only child Kate. Mrs. Harrison sleeps in the Lucknow cemetery, where her husband laid her fully ten years ago, when Kate was a little maid of eight, living in England with her grandmother. A year before our story opens the young lady came out to join her father and keep house for him; and a few months later the household was increased by the doctor's sister, whose soldier husband had fallen a victim to sunstroke, and had left her almost destitute. Father and daughter met as total strangers, for he had last seen her when she was an infant; but they soon grew to understand one another, and if Kate idolised her parent, his affection for her was equally deep and sincere. Miss Harrison ruled the house, and every creature in it, from the pompous, grey-bearded old khansamah, or butler, to the humble individual whose multifarious duties in the cookhouse entitled him to be described on the monthly pay-list as "cook's matey boy." She had no talent for languages, and her knowledge of Hindustani was of the slightest. But if she had not found the road to her native dependents' hearts by the acknowledged route—their mother tongue—she had taken a shorter and more certain path, for every man in the house, stable, or garden would have cheerfully laid down his life for the "Missie Baba." Mrs. Brent, during a long residence in the East, had acquired what, for a lady, was an exhaustive acquaintance with the language, and her intimacy with "bazaar" produce and prices made her a terror to servants whose honesty was doubtful. She was infinitely better qualified to take charge of the house than her neice, but she did not fail to see that her brother was fully satisfied with Kate's method of managing domestic affairs, and wisely accepted a secondary place, whence she only emerged to advise or suggest, when asked to do so. And Miss Harrison, with thoughtful tact, took pains to let her aunt understand that she ruled by her guidance. Thus matters worked smoothly, and no family in Meerut lived in more complete harmony than that in the "Corner Bungalow," as the house was popularly called in the station.

This evening the two ladies, dressed in cool white costumes, are sitting in the drawing-room at afternoon tea. The room is spacious and well furnished. "Calcutta matting" of pale straw colour replaces the soft carpet found in English homes. There are no deep-cushioned chairs and sofas, but the bare canework of the lounges is best adapted for the climate. Draperies of native stuffs adorn and conceal the white-washed walls, and English bric-a-brac and china predominate over Indian productions in the ornamental trifles scattered about on brackets and side-tables. Overhead, the white-draped punkah flaps lazily to and fro, completing the picture of Indian comfort.

"What are you going to do this evening, Kate?" enquired Mrs. Brent of her niece, as she put down her cup. "The carriage has come round."

"I promised to call for Mrs. Arkwright and take her for a turn before going to the band," answered Miss Harrison. "You will come, wo'n't you?"

"I'll come with pleasure," replied her aunt; "but mind,

Kate, if that woman begins again about the ayah my woman got for her, I shall give her a piece of my mind. Mrs. Arkwright is always in hot water with her servants, and its her own fault. She nags at them incessantly, and not a native will stay with her."

"I'm afraid she does bully them a good deal," said Kate, "but she is in trouble just now with her baby, so we must make allowances for her."

"I gave her an infallible recipe for prickly heat," remarked Mrs. Brent severely, "but, of course, I can't make her use it, though I'd like to for the poor baby's sake. Let us go if you are ready."

They entered the smartly appointed landau which was waiting at the verandah steps. The coachman started his horses, the two attendant syces sprang on to the footboard behind, and the equipage rolled through the compound gate and turned down the Mall towards Mrs. Arkwright's house.

"I never can help pitying the English child who has to pass the summer in the plains," said Miss Harrison as she waved her hand to a pale-faced, sickly-looking little boy of about three, who was strapped into a saddle-chair on the back of a diminutive pony. "They ought to establish baby farms up at all the hill stations where parents could trust their children."

"A bold scheme, Kate," laughed her aunt; "it might have advantages for frivolous young matrons, but I'm afraid it wouldn't answer."

"It would be more economical than *that*," said Miss Harrison, indicating a small procession consisting of a child on a pony, led and escorted by a syce and an ayah, in addition to a bearer, whose mission was to look after the other two servants. "How that young autocrat will despise a perambulator when he goes home! Here we are; I wonder if Mrs. Arkwright is ready."

A rather untidy khansamah came forward as the carriage stopped before the house, and approached with a profound obeisance.

"Give the mem-sahib salaams, and tell her we have come," said Mrs. Brent.

The khansamah vanished and gave the message to the bearer, who told the ayah, who went and informed her mistress, this being the way servants "divide work" in the East. An answer saying that the men-sahib would come at once found its way back by the same circuitous method, and had scarcely been delivered when Mrs. Arkwright herself appeared. She was a small, washed-out woman of about three-and-thirty, who had been pretty in her earlier years. Now a woe-begone expression and depressed manner were her distinguishing features, though she retained all her youthful love of gaiety. She climbed into the victoria and took her seat opposite Miss Harrison.

"It's so good of you to come for me, dear," she began; "the Major's gone off pig-sticking again, and the only animal he's left me is that rat-tailed, country-bred pony, which shies at everything it sees. I dare not let the coachman put it in harness."

"When is Major Arkwright coming back?" asked Kate.

"Don't ask me, my dear, for I don't know. He went with some of the 110th men, and took all his camp kit with him. He might think of my feelings sometimes," she added with a sigh; "one never knows what may happen to men who go out pig-sticking in the hot weather—sunstroke, heat apoplexy, goodness only knows what. I grow very anxious about him. Have you heard what date the Artillery have fixed for their dance?" she asked, gliding easily into a more cheerful topic.

"Not yet," replied Kate; "they talk about giving it out on the tennis court, putting down matting to dance on. It would be a change from the club, and ever so much jollier in this weather."

"The officers of the 63rd Punjaubees did that once, about three years ago, at Umballa," said Mrs. Arkwright, pensively. "It was a great success in every way, I remember. Two engagements were announced the very next day."

"Were they direct results of the 63rd's dance?" asked Kate, with an amused smile.

"An open-air dance like that gives such facilities for sitting out, you know," explained Mrs. Arkwright, "and I daresay matters were advanced a little by it. I know Jack Courtney's proposal to the third Miss Gartwood was; he told me so himself. So shy, you know; he could never have said what he wanted in broad daylight, or if there had been the least risk of interruption."

"I see," laughed Kate. "I know the Courtneys; they spent a week with us last cold weather when you were away, and they had two dear babies with them. By the way, how is your little girl?"

"That horrid prickly heat is still bothering her. I was just going to send the klutmugar to buy the stuff you kindly recommended to me the other day, Mrs. Brent," she added, turning to the elder lady, "but Captain Andrews came in to take me for a ride, and I forgot it. I must get it to-morrow."

Mrs. Brent's prescription had been given a week previously, as nearly as that lady could recollect, but she only expressed a hope that it would prove efficacious when it was tried.

"Oh, by the way, Kate," cried Mrs. Arkwright suddenly, as the carriage drew up among a crowd of others round the bandstand in the gardens, "the new man for the 110th is expected to-day. I saw Mrs. Tenterdale this morning, and she told me so."

New arrivals at a "plain's station" are few and far between during the hot season, and their appearance creates almost as much interest as a new curate in a sleepy country town in England. The victoria was soon surrounded by men—civil, military, young, and middle-aged. The more fortunate monopolised Miss Harrison's attention, and the others devoted themselves to Mrs. Brent and Mrs. Arkwright, hoping for a word with the doctor's daughter by-and-by. The majority of the Meerut ladies had flown to Simla or Mussoorie two months ago, and those who remained behind received a double quantity of attention from the slightly diminished circle of men.

A smooth-faced subaltern, who had served his Queen for a period of six months, had been the first to descry Dr. Harrison's carriage, and now reaped the reward of his vigilance in the shape of the empty seat beside Mrs. Arkwright, from which coign of vantage he devoured Kate with all his eyes.

"Well, Mr. Wentworth, what's your news?" enquired Miss Harrison. "How's the pony?"

Mr. Wentworth, who always wore riding gear when not in uniform, and had received the soubriquet of "Fops" from his affection for the boots in which he was said, by young brother officers, to sleep, took the handle of his whip out of his mouth to reply.

"The pony is all right, thank you, Miss Harrison," he said; "and Bairdsley has come."

"He has come, has he?" struck in Mrs. Arkwright. "Now tell us all about him, Mr. Wentworth. Is he a nice man? Does he dance? Is he going to be a useful addition to Meerut?"

"I don't know much about him yet," said Mr. Wentworth; "but I hear that he once rode second in the Grand Military Steeplechase at Cork."

"That is something in his favour, at all events," said Miss Harrison gravely, with a twinkle in her brown eyes. "Can't you gratify our curiosity any further?"

Her own curiosity could not have been very great, for she turned her head, as soon as she had spoken, to greet an elderly man who had come up to the carriage.

"He's a *very* handsome man," said Mr. Wentworth to Mrs. Arkwright, in tones which befitted the importance of the statement; "you will see him here this evening if you wait. The Colonel drove him up to the polo-ground, but they will be back very shortly; it's too dark to play now." The lad heaved a very deep sigh as he spoke; he was one of Miss Harrison's most devoted admirers, and he had seen in Captain Bairdsley another rival; just as though there were not more than enough for him to contend with already!

The party remained at the band, chattering and laughing until "God save the Queen" gave the signal for the assembly to disperse, when Mr. Wentworth vacated the position he had faithfully clung to for half an hour, and went in search of his pony.

"Good night, Miss Harrison!" said her elderly friend, as the carriage moved away. "I'll send it over to your bungalow to-morrow."

"What is that Major Paterson is going to send you, Kate?" asked Mrs. Brent, carelessly.

"A new book—'All Sorts and Conditions of Men.' I saw it criticised in the *Saturday Review* ages ago, and at last a copy has found its way to Meerut."

"I do love a good novel," said Mrs. Arkwright, sympathetically. "I don't know what I should have done without Miss Braddon and Ouida."

"I am tired of them," said Miss Harrison." I have just finished 'The Story of an African Farm.' Major Paterson lent it to me, and I really enjoyed it.

"I liked some parts of it," said Mrs. Arkwright, doubtfully; "but I had to skip a great deal. I couldn't be bothered with it."

"I often think that one of the greatest drawbacks of Indian life is the difficulty we have in getting new music and books. Major Paterson says we are two years behind the world at home in our literature, and I quite believe him."

"A good library would be a blessing," said Mrs. Arkwright, thinking of "Phantom Fortune" and half-a-dozen other works which she had seen advertised during the last three years, but which had never reached Meerut.

"Aren't you going into the club?" she asked, as the coachman walked his horses slowly past the gate for the second time without receiving orders to stop.

"Yes, if you don't care to remain out longer," replied Miss Harrison. "We must call for papa, anyhow, when he has finished his whist."

She gave the coachman the order, and they drove into the grounds, which contained the ladies' rooms of the Meerut Club. Mrs. Arkwright disappeared in the compound, and Mrs. Brent and Kate went to look for the last mail's papers.

Miss Harrison selected one or two periodicals from the table, and having summoned a coolie to pull the punkah, sat down under it in a low garden-chair to read. She found it difficult to keep her attention fixed, for in spite of the jerkily-pulled punkah, the mosquitoes sang round her in swarms, only ceasing their music when her restless hand allowed them an opportunity of settling down to bite. They spared no part they could reach, and attacked her neck, face, and ankles with the relentless perseverance those bloodthirsty insects possess in so terribly marked a degree. Their low "p-i-i-nng" sounded in her ears incessantly, urging her to make fruitless grabs at her invisible foes. The cotton gloves and stockings offered no difficulties to their searching trunks, and presently Kate threw down her paper in despair to rub her bitten feet and hands vigorously.

"The mosquitoes are perfectly awful to-night," she said to Mrs. Brent, who was placidly studying *The Queen*; "do you never feel them, Aunt Agnes"?

"No, Kate; they don't care for such a sun-dried meal as I should make. I've been mosquito-proof for years," replied that lady contentedly.

Kate rested her arms on the verandah railing and stood looking out into moonlight. The Mall was still thronged with carriages moving up and down at a foot pace. Over, on the right, through the trees, she could see into the compound of the club proper, where men—stretched in long arm-chairs, which allowed them to rest their feet at the level of their heads—were smoking under the gallows-like erections from which the punkahs swung. She could not recognise anyone at this distance, but as she idly gazed two figures rose from their seats, and strolled in the direction of the ladies' rooms. Miss Harrison picked up her *Weekly Times*, and returned to her chair; whoever the men might be, they need not find her staring over the end of the verandah as though she were looking out for visitors.

She soon dropped the paper again, however, for the voice she heard was her father's, and she rose to meet him. As she did so, she saw that his companion was a stranger, and guessed that this was the "new man for the 110th."

He was a handsome man of thirty-five or thereabouts, slightly over middle height, and of good figure, clearly cut features, dark hair, and heavy moustache. His bearing was easy and pleasant, and his voice prepossessed Miss Harrison, as it did everyone, in his favour at once.

"My daughter, Captain Bairdsley," said Dr. Harrison. "Kate, let me introduce Captain Bairdsley, who has just arrived at Meerut to join the 110th. Is Agnes here?" he enquired, looking round for his sister.

Mrs. Brent was concealed by her newspaper, from whose columns she was disturbed to make Captain Bairdsley's acquaintance.

Dr. Harrison left him with the ladies, and returned to the club for his evening rubber. He was the hardest worked man in the station, and the hot weather was the busiest time of the year for doctors. The hospitals were full, and not one of the medical staff could call a single hour of the twenty-four his own, and Kate's father went off to whist, cherishing hopes that he might be allowed to have his game without an urgent summons coming to interrupt it.

"Have you been out before, Captain Bairdsley?" asked Miss Harrison, as they seated themselves again.

"Never, Miss Harrison; I only arrived in India for the first time three days ago.

"Do you find the heat very trying, coming straight from home?" asked Mrs. Brent, languidly.

"Not so much as I anticipated," replied Captain Bairdsley, "but is it not the case that the first hot season is the least felt?"

"I think so," answered the lady. "I know that I have been fourteen years in the country, and each succeeding year seems hotter than the last."

(*To be continued.*)

